# Tumor size, stage and grade alterations of urinary peptidome in RCC

**DOI:** 10.1186/s12967-015-0693-8

**Published:** 2015-10-20

**Authors:** Clizia Chinello, Marta Cazzaniga, Gabriele De Sio, Andrew James Smith, Angelica Grasso, Bernardo Rocco, Stefano Signorini, Marco Grasso, Silvano Bosari, Italo Zoppis, Giancarlo Mauri, Fulvio Magni

**Affiliations:** Department of Health Science, School of Medicine, University of Milano-Bicocca (UNIMIB), Via Cadore, 48, 20900 Monza, Italy; Urology Unit, Department of Specialistic Surgical Sciences, Ospedale Maggiore Policlinico Foundation, Milan, Italy; Department of Laboratory Medicine, Hospital of Desio, Desio, Italy; Department of Surgical Pathology, Cytology, Medical Genetics and Nephropathology, Azienda Ospedaliera San Gerardo, Monza, Italy; Department of Medicine, Surgery and Dental Sciences, Pathology Unit, IRCCS-Policlinico Foundation, Mangiagalli and Regina Elena, University of Milan, Milan, Italy; Department of Informatics, Systems and Communication, University of Milano-Bicocca, Milan, Italy

**Keywords:** Mass spectrometry, Proteomics, Urine, Renal cell carcinoma, Tumour size, Tumour progression, Stage, Grade, Cancer, Peptidomics

## Abstract

**Background:**

Several promising biomarkers have been found for RCC, but none of them has been used in clinical practice for predicting tumour progression. The most widely used features for predicting tumour aggressiveness still remain the cancer stage, size and grade. Therefore, the aim of our study is to investigate the urinary peptidome to search and identify peptides whose concentrations in urine are linked to tumour growth measure and clinical data.

**Methods:**

A proteomic approach applied to ccRCC urinary peptidome (n = 117) based on prefractionation with activated magnetic beads followed by MALDI-TOF profiling was used. A systematic correlation study was performed on urinary peptide profiles obtained from MS analysis. Peptide identity was obtained by LC–ESI–MS/MS.

**Results:**

Fifteen, twenty-six and five peptides showed a statistically significant alteration of their urinary concentration according to tumour size, pT and grade, respectively. Furthermore, 15 and 9 signals were observed to have urinary levels statistically modified in patients at different pT or grade values, even at very early stages. Among them, C1RL, A1AGx, ZAG2G, PGBM, MMP23, GP162, ADA19, G3P, RSPH3, DREB, NOTC2 SAFB2 and CC168 were identified.

**Conclusions:**

We identified several peptides whose urinary abundance varied according to tumour size, stage and grade. Among them, several play a possible role in tumorigenesis, progression and aggressiveness. These results could be a useful starting point for future studies aimed at verifying their possible use in the managements of RCC patients.

**Electronic supplementary material:**

The online version of this article (doi:10.1186/s12967-015-0693-8) contains supplementary material, which is available to authorized users.

## Background

Renal cell cancer (RCC) accounts for about 4 % of all adult cancers and is the most dangerous of all urological malignancies with a median survival time of about 13 months, with less than 10 % of patients surviving more than 5 years. There are about 210,000 new cases and 100,000 deaths diagnosed worldwide every year, and the number of patients is increasing rapidly [1]. About 30 % of patients already have metastasis at the time of diagnosis and 30–40 % of patients with localized kidney cancer will have a recurrence [2]. Although partial or total surgical resection are the gold standard treatments for patients with localized RCC, approximately 20–40 % of them will have progression during one-year follow-up period. Neither chemotherapy nor radiation therapy is effective for the patients with metastases [1].

Over the last decade, most of kidney tumours are revealed incidentally during unrelated clinical investigation, owing to the widespread use of abdominal imaging, including ultrasonography and computed tomography. Moreover, laboratory examinations accompanied by urine analysis in search for haematuria are prompted for every suspicion of RCC [3]; however, there are still no other cancer type- or stage specific biomarkers appropriate for clinical usage in urine analysis [4]. On the other hand, the increase of RCC incidence is also associated with the rise in incidental detection of localized small renal masses. About 20 % of small masses are benign and they are likely to have pathological characteristics of low Fuhrman grade and clear cell type. In addition, small renal masses are increasingly detected in elderly patients who probably have comorbidities and are a high-risk group for active treatment like surgery [5]. Consequently, indications for watchful-waiting in small renal kidney cancers are consistently expanding [6]. As the management possibilities are enhancing, the current role of renal mass biopsy is also increasing. Thus, despite the associated risks (bleeding, tumor seeding, etc.), renal mass biopsy is recommended for active surveillance in most cases except in watchful-waiting candidates, in patients with imaging or clinical characteristics indicative of pathology as syndromes and in cases whereby conservative management is not contemplated [7].

Rapid progress has been made to understand tumorigenesis, but the key events leading to the dysfunction of the control of cellular proliferation still remain unclear. Since alterations of these regulatory processes can be caused not only by altered gene expression but also by proteins, proteomics investigations are of special importance to detect patterns of disease-associated protein alterations. Recently, we and other researchers used these approaches to identify differentially expressed proteins by comparing non-neoplastic and neoplastic kidney tissue as possible biomarkers [8–10]. Moreover, biomarker discovery for RCC is not only feasible in the tissue proteome but also in urinary peptidome. In fact, peptidomics analysis has detected the presence of clusters of urinary peptides able not only to differentiate RCC patients from controls [11] but even benign from malignant kidney solid masses [12].

Following surgery, TNM (Tumour-Node-Metastasis) staging and Fuhrman grade as well as performance status and serum markers (haemoglobin, calcium, lactate dehydrogenase, platelets, neutrophils and C-reactive protein) are the most used factors applied so far for predicting tumour progression and recurrence [13]. Many authors have therefore investigated the role of prognostic biomarkers attempting to improve patient’s risk stratification, and better predict disease recurrence and associated mortality [13, 14]. Quite recently, a predictive model based on the following independent prognostic factors has been published by Tosco et al. [15]: primary tumour T stage ≥3, primary tumour Fuhrman grade ≥3, nonpulmonary metastases, disease-free interval ≤12 mo and multiorgan metastases. Considering that cancer progression is reflected by tumour stages, Junker et al. have examined neoplastic tissues from kidneys belonging to different stages and grades as well as the non-neoplastic specimen counterparty [10]. In particular, they have studied the proteome of kidney tissue of 27 patients (pT1, pT2, pT3) and 9 adjacent normal tissue by 2D-DIGE, identifying several proteins correlating with pT. Lebdaj et al. found a significant association between expression of transforming growth factor beta-induced (TGFBI) in tissue with tumour category pT3-pT4, Fuhrman grades III and IV and tumour mass size >4 cm [16]. Moreover, Morrisey et al. have observed a correlation between the neoplasm size and the pre-nephrectomy urinary levels of AQP1 and PLIN2. A correlation was found also for both markers with tumour stage but not with grade [17]. The identification of biomarkers predicting treatment response could avoid unnecessary costs, prevent side effects, and help in choosing optimal patient management. Recently, progress in the chemotherapeutic treatment of renal cell carcinomas has been achieved with the development of targeted molecular therapies [16]. Currently, several promising molecular biomarkers have been found, but none of them have been introduced in clinical practice. Consequently, the most powerful tool for predicting tumour aggressiveness still remains stage, size and grade [16]. However, as it stands, no systematic investigation has been carried out to highlight if part of the urinary peptidome not only suggests the presence of a tumour mass in kidney [11, 12] but may also reflect its progression. Therefore, in this study we investigated the urinary peptidome to search for and identify peptides with a urinary expression alteration according to pT, tumour dimension and grade to be employed as a possible starting point for future more specific studies aimed at ccRCC management.

## Methods

### Chemicals and standards

Profiling Kit 1000 C8-MB, α-cyano-4-hydroxycinnamic acid (CHCA), Protein Calibration Standard I (ProtMix I) and Peptide Calibration Standard II (PepMix II) were provided by Bruker Daltonics GmbH (Bremen, Germany).

### Urine collection and handling procedure

Urine samples were collected from patients the day before surgery at “Ospedale Maggiore Policlinico” Foundation (Milan, Italy), San Gerardo Hospital (Monza, Italy) and those from healthy volunteers at Desio Hospital (Desio, Italy). All subjects had signed an informed consent prior to sample donation and analyses were carried out in agreement with the Declaration of Helsinki. Study protocols and procedures were approved by the local ethic committee (U.O. Comitato di Etica e Sperimentazione Farmaci Direzione Scientifica Fondazione IRCCS Ca’Granda Ospedale Maggiore Policlinico, Milano and Comitato Etico Azienda Ospedaliera San Gerardo, Monza, Italy). Second morning midstream urine was collected in sterile urine tubes (Anicrin s.r.l., Italy) [18].

Prefractionation of urine samples was performed as already described [12]. Briefly, 40 µL of the urine sample was incubated for 1 min in a magnetic separator with 80 µL of binding buffer and 5 µL of RPC8 magnetic beads. The beads were then washed twice with 45 µL and once with 30 µL of washing solution. Finally, the peptides were released with 10 µL of elution solution (50 % Acetonitrile in water). A small amount of the eluates was used for the automated spotting onto an AnchorChip™ target (Bruker Daltonics, Germany).

In addition, two pools were obtained by mixing 40 µL of urine of each subject and used for peptide identification. The two pools were manually purified with magnetic beads following the same steps and proportion described for the automatic procedure.

### MALDI-TOF peptide profiling

The analysis of purified urine samples was performed in linear mode (MALDI-LM) and reflector mode (MALDI-RM) using an UltrafleXtreme™ MALDI-TOF/TOF instrument (Bruker Daltonics, Germany) [19].

Multiple spectra comparison was performed with ClinProTools™ Software v. 2.2 (Bruker Daltonics, Germany) after normalization. Statistical elaboration was conducted on the mean spectrum generated from each subject dataset. A S/N threshold of 3 and zero level integration type were used as parameters in order to obtain a list of peaks with their area, calculated on the total average spectrum.

### Expression profile analysis and statistical analysis

Statistical analysis was conducted as already described [12]. Initially normality and homogeneity of variance (Shapiro–Wilk’s, Barlett and Leven’s test for normality and homogeneity testing) were evaluated, followed by suitable parametric or non-parametric tests for groups comparisons (Equal Variance *t* test for Normal Data with Equal Variances, Unequal Variance (Welch) t-test for Normal Data with Unequal Variances, Mann–Whitney U-test (Wilcoxon) for Non-normal Data with Equal Variances and Kolmogorov–Smirnov test for Non-normal Data with Unequal Variances). Peptide urinary expression variation according to clinical data was computed using Spearman rank-order correlation (Spearman’s rho). All tests were applied using 0.05 as the significance level.

### Peptide sequencing by nLC–ESI–MS/MS

Identity of the endogenous peptides present in RPC8 enriched fractions were assessed by nLC–ESI–MS/MS analysis of the ccRCC patients urine pool (n = 80) prepared as previously described [19]. Briefly, before injection MB fractionated samples underwent a purification step from salts and beads performed using Ziptip™ μ-C18 Pipette Tips (Millipore Corp, Bedford, MA, USA) as already reported [19]. Desalted fractions were chromatographically separated into a Dionex UltiMate 3000 rapid separation (RS) LC nano system (Thermo Scientific, Germany) and analysed with an online-coupled Impact HD™ mass spectrometer (Bruker Daltonics, Germany).

Peptides were loaded onto a µ-precolumn (Dionex, Acclaim PepMap 100 C18, cartridge, 300 µm i.d. × 5 mm, 5 µm), followed by a multistep 360 min gradient at a flow rate of 300 nl/min on the analytical 50 cm nano column (Dionex, 0.075 mm ID, Acclaim PepMap100, C18, 2 µm). A ramp from 4 to 35 % in 245 min of mobile phase B (0.1 % FA/80 % CHCN) was used. The column was connected to a nanoBooster CaptiveSpray™ (Bruker Daltonics). The mass spectrometer was operated in the data-dependent-acquisition mode to automatically switch between full scan MS and MS/MS acquisition in CID mode (N_2_ was used as collision gas). The number of precursor ions was automatically adjusted to fit into a fixed cycle time of 5 s, and IDAS (Intensity Dependent Acquisition Speed) and RT2 (RealTime Re-Think) functionalities were applied. The tune parameters were set as already described [12] in order to better assist the fragmentation of larger endogenous peptides.

XML peaklists were processed using an in-house Mascot search engine (v2.4.1). Database searching was restricted to human Swiss-Prot (accessed Apr 2015, 548,208 sequences; 195,282,524 residues). Mass tolerances were set at 20 ppm for MS and 0.05 Da for MS/MS. No enzyme and any fixed modification was set in the search parameters. Acetyl (N-term), Acetyl (K), Deamidation (NQ), Oxidation (HW), Oxidation (M) were set as variable modifications in the Mascot search parameters. Mascot score thresholds and decoy database were used as peptide level filters of peptide significance (FDR <5 %).

## Results

### Clinical data and study design

Urine collected from 117 clear cell RCC patients (ccRCC) (72 men, 45 women) were used in the present study. Fisher test did not reveal any gender dependence in the studied cohort. The mean age for patients was 64.05 with a range of 33–87 years. Patients were classified according to the 2009 TNM system classification and their clinical characteristics are described in Table 1 [12]. Histological analysis was performed on patients based upon the Fuhrman grading system, sarcomatoid and cystic differentiation, tumour necrosis, microvascularity and urinary infiltration. Dimension of the tumour mass was in the range from 1.5 to 18 cm. RCC patients were divided in four groups according to pT (group 1 = pT1a; 2 = pT1b; 3 = pT2a and 4 = pT ≥ 2b) or Grade values (1, 2, 3 and 4). Urine samples collected from 137 healthy subjects (81 men, 56 women) were used for statistical analysis [12].

**Table 1:** Patients clinical characteristics according to the 2009 TNM (tumour-node-metastasis) system classification

	No. of patients
ALL	117
Mean ± SD age at diagnosis	64.05 ± 11.16
Median age at diagnosis (range)	33–87
Tumor mass in cm (range)	1.5–18
Gender	
Males	72
Females	45
Staging	
pT1a	45
pT1b	39
pT2a	23
pT2b	1
pT3a	9
pT3b	0
Grade	
1	6
2	79
3	20
4	3

### Peptide urinary expression altered according to clinical data

To investigate the possible association between urinary levels of peptides and tumour size, pT and grade, statistical analysis was applied to the peptidomic profiles of the ccRCC patients. Peptides with a urinary expression correlating with age were not considered [12].

#### Urinary peptides varied depending on tumour size

Fifteen peptides showed a statistically significant variation according to tumour size (Additional file 1: Table S1A). Almost all of these peptides also have a statistically different expression from control subjects (Additional file 1: Table S1A, second column). Interestingly, the alteration was negative for three of them (range from −0.307 to −0.312) and positive for the other twelve (range from 0.31 to 0.514).

#### Peptide urinary expression altered according to pT

Twenty-six peptides displayed a statistically significant variation between their urinary concentration and pT (Additional file 2: Table S2A). Fifteen of these peptides were differentially expressed in RCC compared with controls (Additional file 2: Table S2A, second column). Notably, only five of them showed a negative alteration (range from −0.202 to −0.326) while for the other twenty-one the variation was positive (range from 0.203 to 0.27).

#### Urinary peptides altered according to Grade

Only five peptides showed a statistically significant alteration between their urinary concentration and grade (Additional file 3: Table S3A). Four peptides were significantly differentially expressed between patients and controls (Additional file 3: Table S3A, second column). Only one of them showed a positive variation while for the other four it was negative with a value of about −0.2.

### Urinary peptides differentially expressed according to histological data

To investigate the possible alterations of the urinary levels of peptides according to pT and Grade, statistical analysis was applied to the peptidomic profiles of the RCC patients divided into four groups.

**Fig. 1:**
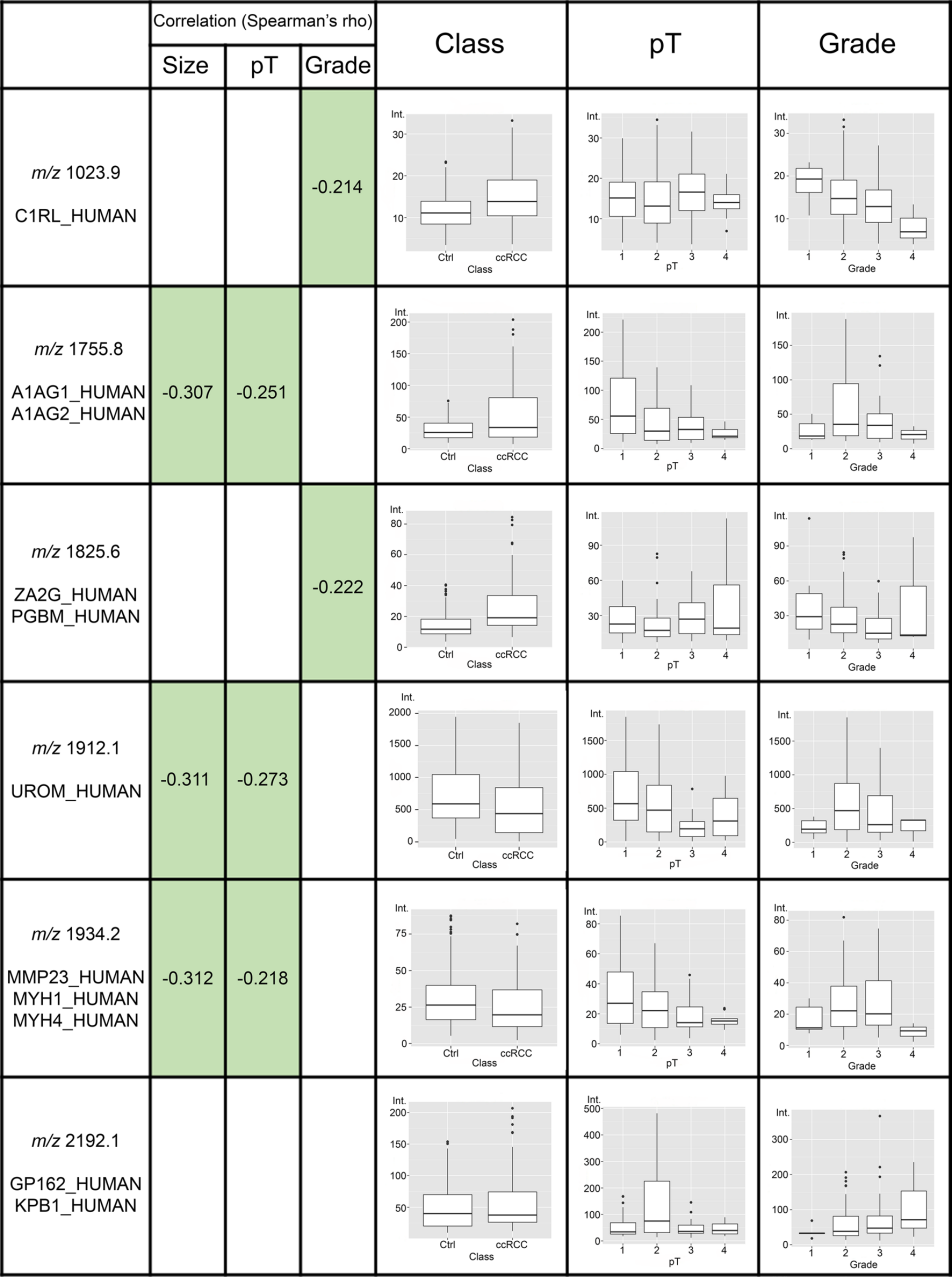
Box-plot of identified peptides (below 2500 m/z) that resulted statistically varied according to class (CTRLs vs ccRCC) pT or grade. The possible significant correlation with tumor size, TNM stage and Fuhrman grade is reported

**Fig. 2:**
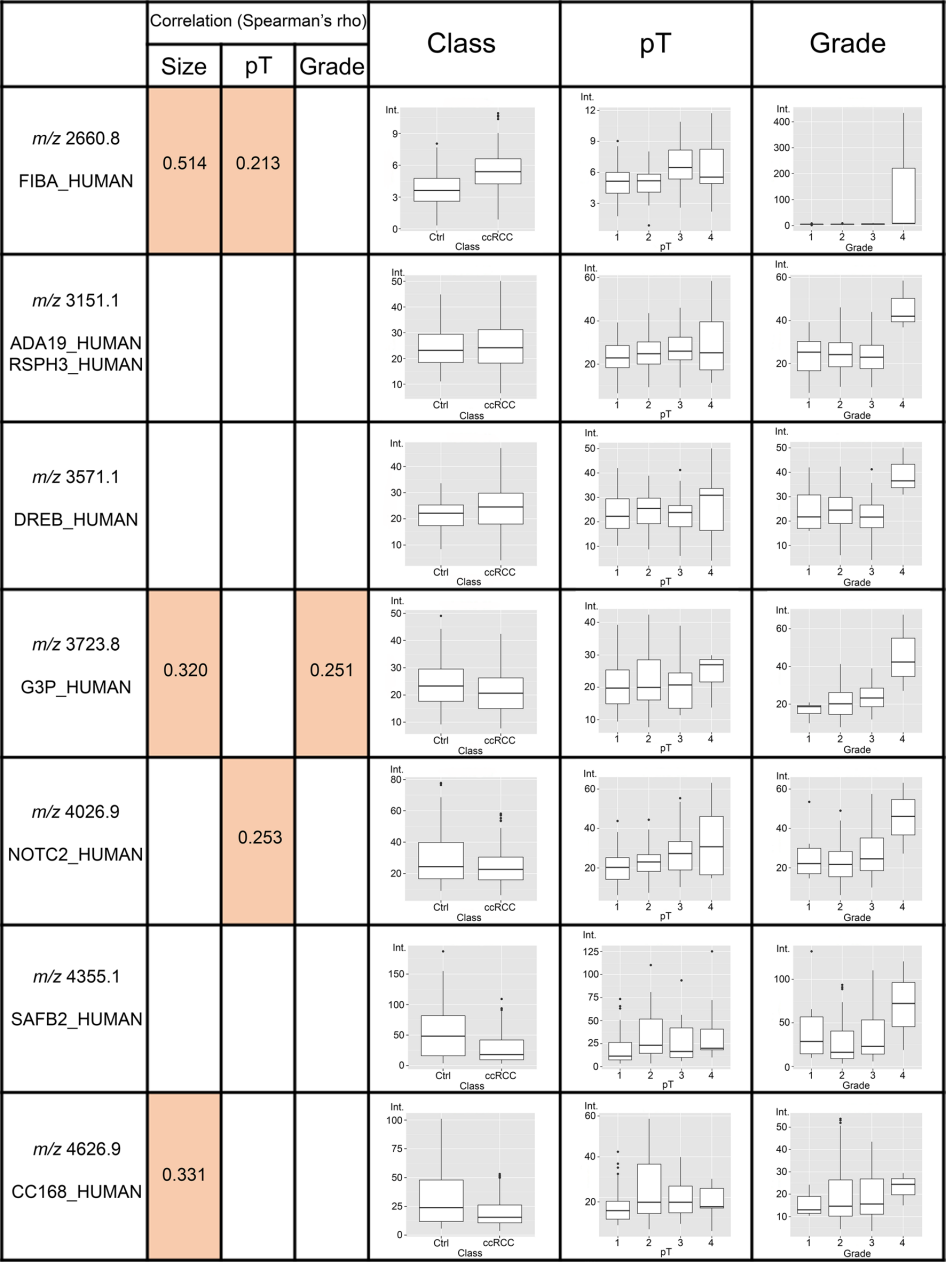
*Box-plot* of identified peptides (above 2500 m/z) that resulted statistically varied according to class (CTRLs vs ccRCC) pT or grade. The possible significant correlation with tumor size, TNM stage and Fuhrman grade is reported

Fifteen peptides were observed to have urinary levels statistically different in patients at different pT values (Additional file 2: Table S2B, Figs. 1, 2), and nine of them were significantly also varied in patients compared with control subjects (Additional file 2: Table S2B, second column). Furthermore, considering only the comparison between patients with tumour stage pT1a and pT1b, fourteen peptides showed a statistically significant alteration (Additional file 4: Table S4).

On the contrary, nine peptides (Additional file 3: Table S3B, Figs. 1, 2) showed a statistically significant difference between patients having a tumour at different grades, with seven of them also varied in patients *versus* healthy subjects (Additional file 3: Table S3B, second column).

### Peptide identification

Identity of the urinary peptides in the C8-magnetic beads pre-purified fractions was obtained by nanoLC-ESI–MS/MS. Several peptides could be identified and among them, fifteen signals having a variation linked to clinical data were recognized (Additional file 5: Table S5).

Six ions present in MALDI-LM profiles and not in MALDI-RM spectra were matched with peptide sequences identified in ESI–MS/MS (average Mass Measurement errors for LM: 222 ± 411.1 ppm): the ion at m/z 3571.1 was recognized as the fragment _362_SQPPPLPPPPPPAQETQEPSPILDSEETRAAAPQ_395_ of the DREB protein; the ion at 3723.8 as the _51_ STHGKFHGTVKAENGKLVINGNPITIFQERDPSK_84_ fragment originated from G3P_HUMAN; the ion at m/z 4355.1 as the acetylated form of the _386_EEKDIKPIIKDEKGRVGSGSGRNLWVSGLSSTTRATDLK_424_ peptide belonging to the SAFB2 protein; the ion at m/z 4626.9 as the _868_HEVKTIDMRFRIHCQEARISPMSHILNAKELVLNINKLE_906_ fragment from CC168 protein. The remaining nine of the fifteen signals were also detected in MALDI-RM profiles, strengthening the accuracy of identity assignment (average Mass Measurement errors for RM: 36.7 ± 81.3 ppm).

In particular, the ion at m/z 1755.8 was identified as a peptide deriving from A1AGx, which is common to both A1AG1 and A1AG2 protein isoforms. The ion at m/z 1934.2 could be generated from two different peptides, whose masses could not be unequivocally assigned observing the isotope cluster obtained in MALDI-RM: the fragment _1152_ERLEEAGGATSAQIEMNK_1169_ deriving from the MYH1_HUMAN/MYH4_HUMAN or/and the fragment _40_LGAPAVPAWSAAQGDVAALGL_60_ originating from the MMP23 protein. Similarly, the ion at *m/z* 1825.6 could be ascribed to amino acid sequence _59_FRYNSKDRKSQPMGL_73_ of ZA2G_HUMAN and/or to _4283_VSEDPINDGEWHRVTA_4298_ peptide of PGBM_HUMAN. For two of the thirteen signals (m/z 2192.1 and m/z 3151.1), the resolving power of MALDI-RM and the low mass difference between different amino acid sequences identified by ESI MS/MS have not allowed the specific contributes to MALDI-LM peak to be unambiguously distinguished.

Three signals were attributed to the same specific amino acid sequences reported in our previous papers [12, 18]. The ion at m/z 1912.1 was already identified as an UROM fragment [18], the ion at m/z 2192.1 as two possible peptides belonging to GP162_HUMAN and/or KPB1_HUMAN [12], while the ion at m/z 2660.8 was unambiguously identified as a fragment from FIBA, also by MALDI MS/MS analysis [12].

## Discussion

Renal cell carcinoma, the most common type of human kidney cancer, is increasing in incidence and it is the most lethal genitourinary malignancy. Several studies aiming at biomarker discovery in RCC have been reported in recent years. Most of them deal with the search of markers for early diagnosis, for prognosis and for the prediction of patients’ response to therapy. Several proteins correlating with the pT were observed when comparing tissue from patients at different RCC progression stages [10]. A significant association between TGFBI tissue expression with this progressive neoplasm at different stages and with its diameter was very recently reported [16]. Moreover, urinary levels of AQP1 and PLIN2 were observed to be correlated with the tumour size and stage but not with grade [17]. However, despite these promising studies, none was successfully able to predict RCC aggressiveness, and, up to now, tumour size and growth rate are still the most used prognostic factors. In addition, currently there is only limited literature addressing urine markers for RCC. This is noteworthy especially if we consider the benefits related to the possibility of following localized tumours or monitoring drug-based therapy results by simply analysing tumour-specific markers in an easily accessible biofluid such as kidney excretory product [20, 21].

In this study, we systematically investigated the urinary peptidome of a large cohort of RCC patients in order to highlight possible features varying according to tumor characteristics: size, stage and grade. To preserve the homogeneity of the samples, we included only clear cell RCC. Statistical data elaboration was focused on data derived from urine samples pre-fractionated using C8 magnetic beads followed by MALDI profiling analysis. Previously, the application of this high-throughput approach allowed us to build clusters of urinary peptides with high diagnostic performances [12].

Through this strategy, we have observed that the urinary abundance of fifteen and twenty-six peptides varies depending on size and stage, respectively (Additional file 1: Table S1A, Additional file 2: Table S2A), and among them eight were shared and showed a consistent correlation trend. On the other hand, only few signals displayed a significant alteration according to Fuhrman grading system (Additional file 3: Table S3A). Even if Furhman grade is one of the most widely accepted histological prognostic factors [22], it has to be considered that some controversial aspects related to the low accuracy due to the grade heterogeneity within the same tumor, and to the interobserver and intraobserver variability in assigning tumor grade are present [23].

Moreover, most of these peptides were higher or lower represented (p < 0.05) in urine of RCC patients compared to control subjects (Additional file 1: Table S1A, Additional file 2: Table S2A, Additional file 3: Table S3A). Some of them have shown significantly varied urinary concentration according to pT or grade (Additional file 2: Table S2B and Additional file 3: Table S3B, Figs. 1, 2) and also at early stages pT1a and pT1b (Additional file 4: Table S4).

One of the major advantages of MALDI profiling strategy is that signals do not need any prior knowledge about their identity in order to allow their use as biomarkers. Nevertheless, the identification of endogenous peptides could increase the biological insight, exploring the function and the regulation of bioactive molecules and degradome products. Furthermore, the lack of identity carries drawbacks for their possible translation into clinical routine laboratories. We could identify fifteen peptides whose urinary expression was significantly varied with tumour size, stage and grade (Additional file 5: Table S5, Figs. 1, 2). Two of the signals were recognized as fragments belonging to highly abundant proteins in urine, FIBA and UMOD. However, the other identified peptides might be attributed to proteins playing a possible role in tumorigenesis, progression and aggressiveness.

In particular, the A1AGx protein, known as a1-acid glycoprotein (AGP), is the major member of the APP family, and its serum concentrations increase during acute-phase reactions [24]. In addition, an increased APP response was observed to be associated with reduced survival rate, independently from stage of malignant disease, including lung, pancreatic, renal, and colorectal cancer and lymphoma [24]. Serum AGP levels in the patient group (esophagus, gastric, colorectal, lung, hepatic, pancreatic carcinoma) did not show any statistical difference according to tumour size, stage, and clinical status [25]. However, patients with advanced or recurrent disease and/or metastasis had higher serum levels of AGP. These findings suggest that its serum levels may not be necessarily related to disease progression [25]. Likewise, we found an increase of the urinary levels of the AGP fragment compared to controls, especially in the early stage pT1a, that decline continuously from pT1 to pT3. A negative variation with tumour size was also observed. Alteration of the A1AG1 non-glycosylated forms levels with the pT was also reported in tissue of RCC patients [10]. The authors investigated tissue from 9 patients at pT1, pT2 and pT3 and they observed a down-expression of this protein in patients at pT1 and pT2 but not at pT3.

NOTCH2 belongs to the Notch family, including NOTCH1, NOTCH2, NOTCH3, and NOTCH4 receptors. Their extracellular domain after the binding with specific ligands induce an alteration of the gene expression. The clear role of the notch family in RCC is still unknown. However, a down-regulation of the expression of Notch receptors and of Notch signalling was reported, thus suggesting a possible role in the progression of renal cell carcinoma [26]. Recently, a specific role for each of the four Notch receptors in RCC has also been shown [27]. These authors showed a reduced expression of Nocth1 correlating with an increase of the Fuhrman grade and tumour size while Notch3 and 4 receptors were directly correlated with tumour size. Therefore, they suggested a possible role of NOTCHs in tumorigenesis of RCC. Our data shows that Notch2 is also involved in RCC progression. In fact, even if the urinary NOTCH2 is reduced in patients compared to controls, a positive variation and an increase of its urinary fragment levels from pT1 to pT3 were observed.

Disintegrin and metalloproteinase 19 (ADAM19) is a cell surface glycoprotein belonging to the ADAM family. These proteins are known to be involved in cell adhesion, fusion, and migration and they have a role in cancer cell proliferation and progression [28]. ADAM19 was described as up-regulated in human brain tumours and correlating with its invasiveness. Moreover, high expression of ADAM19 was also associated with lung and kidney inflammatory and fibrotic processes [29]. An overexpression of ADAM19 in endometrial carcinoma and its correlation with the progression and prognosis [30] as well as in renal cell carcinomas has been reported [31]. No statistical difference in its abundance was observed in ccRCC patients but an increment was noticed in urine of ccRCC at Grade 4. However, the functions of ADAM19 in cancers still remain to be elucidated.

Complement C1r subcomponent-like protein (C1RL) causes the proteolytic cleavage of HP/haptoglobin. The complement system is well known to be involved in many immune complex-mediated kidney diseases: an excessive activation of the alternative complement pathway is associated with autosomal dominant polycystic kidney disease (ADPKD) progression. In fact, levels of the specific complement components such as CFB, SERPING1 and C9 were found to be increased while C1RL, CD55 and CD59 levels were decreased in urine of ADPKD patients. We found higher urinary levels of CRL in RCC patients than in control subjects that slowly decreased from G1 to G4. However, its role in cancer is not known and its detection in tissue is still under investigation [32].

We observed a statistically significant increase of urinary levels of a peptide at m/z 1826 in ccRCC patients compared to control subjects negatively varying with grade. The identity of this signal could have originated either from PGBM or from ZAG proteins.

Basement membrane-specific heparan sulfate proteoglycan core protein (PGBM), belonging to heparin sulfate proteoglycan (HSPG) family, has an important role in vascularization. It has been shown that heparanase is highly expressed with a positive correlation with tumour stage and poor prognosis in RCC [33]. Moreover, HSPGs have been reported in many metastatic tumours, and their expression was observed in a variety of malignant tumours, showing correlations with malignant phenotype [34]. Higher expression of heparanase in ccRCCs than in non-ccRCCs correlating with stage was shown by immunostaining and RT-PCR [35]. Furthermore, specific silencing of heparanase mRNA expression (786-O and Caki-2 cells) with small interfering RNA (siRNA) inhibited the invasiveness capabilities of these cells in vitro [35]. Elevated heparanase expression was also shown to be an independent indicator of disease-specific survival [35, 36]. These findings suggest an important role of heparanase in invasion and metastasis and silencing of the gene could represent a potential therapeutic target in ccRCCs.

Zinc-alpha-2-glycoprotein (ZA2G, AZGP1, ZAG) is a protein associated with lipid mobilization, a process that is also regulated by mTOR signaling. A down expression of the ZA2G was observed in the tissue of patients affected by hepatocellular carcinoma (HCC) [37] and it was associated with a poor overall survival, worst disease-free survival, relapse-free survival and distant metastatic progression-free survival. Moreover, ZA2G protein was also found to be lower in 57 patients with a rising of prostate specific antigen after surgery and not in the other 32 patients [38]. ZAG expression was inversely correlated with Gleason pattern and correlated with a favourable outcome.

MMP23 is a member of matrix metalloproteinase family (MMP23A, MMP21, MMP23B, MMP22) that participates in many aspects of tumour growth and metastasis. Altered MMP23 expression has been observed in prostate adenocarcinoma, multiple myeloma, synovial sarcoma and in colorectal cancers [39–43]. We observed that its urinary levels are lower in RCC patients compared to controls and have a negative alteration depending on tumour size and pT.

Drebrin (DREB) has been recently reported to be over-expressed in human metastatic colon adenocarcinoma cell line HCT-116 [44]. We also detected an increase of its urinary levels in ccRCC, especially at grade 4. A possible role in tumour cell migration and invasion for this protein has been shown in glioma. A constant overexpression of drebrin in U87 cells caused an alteration in cell morphology with an increased invasiveness while a silencing of drebrin gene expression decreases the invasion and the migration [45].

Glyceraldehyde-3-phosphate dehydrogenase (G3P) is implicated in nuclear functions such as transcription, RNA transport, DNA replication and apoptosis. Down-regulation of its expression was observed in colorectal cancer [46] while it was over-expressed in human cancer cell lines [47]. Moreover, increased expression of GAPDH enhanced aggressiveness and vascularization of non-Hodgkin’s lymphoma [48].

SAFB1 (scaffold attachment factor B1) and a second family member Scaffold attachment factor B2 (SAFB2) are multifunctional proteins implicated in a variety of cellular processes including cell growth, apoptosis and stress response. A possible role for SAFBx as tumour suppressors has also been suggested [49]. There is numerous evidence that SAFB1/SAFB2 have a contribution in cancer progression. SAFB1/SAFB2 may have more complex implications in cellular functions, such as RNA processing and metabolism, that could potentially affect various signalling pathways in cancer [49]. We observed low urinary levels for SAFB2 in RCC, patients especially at grade 4. Low SAFB protein levels were also suggested as possible predictors of poor prognosis of breast cancer [50].

There is a critical need for trials aimed at better defining the progression of renal masses as well as finding robust indicators of patient outcomes, such as overall survival and disease specific survival. These studies should include either hypothesis generating or hypothesis testing of laboratory tools, tissue based or circulating biomarkers [51]. Moreover, the American Urological Association guidelines state that patients undergoing follow-up for treated or observed renal masses should undergo basic laboratory testing and, depending on the risk, imaging (US, CT or MR) every three months in high risks or yearly in low risks. However, potential adverse effects and cost should also be take into account. Recent attention has been paid to the cumulative radiation exposure of the population attributable to the widespread and increasing use of CT scanning. For MRI, which does not involve the use of ionizing radiation, the prime adverse effect to consider is the development of nephrogenic systemic fibrosis (NSF) due to IV gadolinium administration. No prospective validation currently exists for the use of common laboratory parameters in the early detection of metastases or both in the staging and monitoring of patients with renal cell carcinoma following treatment for recurrence. Moreover, the finding of a biological aggressiveness marker is also useful considering its role in the active surveillance of the appropriately selected small renal mass, limiting adverse health outcomes. In this context, it is desirable to gain easily accessible, not invasive molecular indicators, which correlate with consolidated progression factors, such as tumor size, staging and grade. Moreover, an understanding of the complex molecular alterations involved in the development and progression of RCC could enable development of immunohistochemical and immunoenzymatic diagnostic tools and also open the doors for experimental targeted therapies.

## Conclusions

In this study, we highlight a number of peptides whose urinary expression is altered depending on tumor size, pT and grade. Among them, several play a possible role in tumorigenesis, progression and aggressiveness, enriching the molecular scenario of RCC development. These results could be a useful starting point for future studies aimed at verifying their urinary levels by immunoenzymatic assays (i.e. ELISA test) and the resulting possibility for some of them to be implemented in progression algorithms for risk stratification of ccRCC patients. Moreover, their urinary changes could also be useful to the “watch-and-wait approach” for monitoring small renal masses and could find a possible role in management follow-up strategies (e.g. residual tumors).

## Additional files

10.1186/s12967-015-0693-8  Peptides with a statistically significant (p < 0.05) urinary abundance (m/z area) according to the tumour mass (cm). Up/down refer to the over/under representation of the peptide in ccRCC patients compared to control subjects.
10.1186/s12967-015-0693-8  **A)** Peptides with a statistically significant (p < 0.05) urinary abundance (m/z area) according to the pT. **B)** Peptides with a statistical significant (p < 0.05) urinary abundance (m/z area) compared to CTRLs and at different pT (1 = pT1a; 2 = pT1b; 3 = pT2a and 4 = pT > 2b). Up/down refer to the over/under representation of the peptide in ccRCC patients compared to control subjects
10.1186/s12967-015-0693-8  **A)** Peptides with a statistical significant (p < 0.05) urinary abundance (m/z area) according to the Grade. **B)** Peptides with a statistically significant (p < 0.05) urinary abundance (m/z area) compared to CTRLs and at different Grade (1, 2, 3 and 4). Up/down refer to the over/under representation of the peptide in ccRCC patients compared to control subjects.
10.1186/s12967-015-0693-8  Peptides with a statistically significant (p > 0.05) urinary abundance (m/z area) between pT1a and pT1b. Up/down refers to the over/under representation of the peptide in pT1b compared to pT1a patients.
10.1186/s12967-015-0693-8  Identification of MALDI signals varied according to tumour size, pT or grade. LM = linear mode, RM = reflector mode, PTM = possible hypothetical modification predicted by Mascot.
